# Nodal Promotes the Self-Renewal of Human Colon Cancer Stem Cells via an Autocrine Manner through Smad2/3 Signaling Pathway

**DOI:** 10.1155/2014/364134

**Published:** 2014-02-17

**Authors:** Yuehua Gong, Ying Guo, Yanan Hai, Hao Yang, Yang Liu, Shi Yang, Zhenzhen Zhang, Meng Ma, Linhong Liu, Zheng Li, Zuping He

**Affiliations:** ^1^State Key Laboratory of Oncogenes and Related Genes, Stem Cell Research Center, Renji Hospital, Shanghai Jiao Tong University School of Medicine, 1630 Dongfang Road, Shanghai 200127, China; ^2^Department of Urology, Shanghai Human Sperm Bank, Renji Hospital, Shanghai Jiao Tong University School of Medicine, 145 Shandong, Shanghai 200001, China; ^3^Shanghai Key Laboratory for Assisted Reproduction and Reproductive Genetics, Shanghai 200001, China; ^4^Shanghai Key Laboratory of Reproductive Medicine, Shanghai 200025, China

## Abstract

Colorectal cancer is one of the most common and fatal tumors. However, molecular mechanisms underlying carcinogenesis of colorectal cancer remain largely undefined. Here, we explored the expression and function of Nodal in colon cancer stem cells (CCSCs). Nodal and its receptors were present in numerous human colorectal cancer cell lines. NODAL and ALK-4 were coexpressed in human colon cancerous tissues, and NODAL, CD24, and CD44, markers for CCSCs, were expressed at higher levels in human colon cancerous tissues than adjacent noncancerous colon tissues. Human CCSCs were isolated by magnetic activated cell sorting using anti-CD24 and anti-CD44. Nodal transcript and protein were hardly detectable in CD44- or CD24-negative human colorectal cancer cell lines, whereas Nodal and its receptors were present in CCSCs. Notably, Nodal facilitated spheroid formation of human CCSCs, and phosphorylation of Smad2 and Smad3 was activated by Nodal in cells of spheres derived from human CCSCs. Collectively, these results suggest that Nodal promotes the self-renewal of human CCSCs and mediate carcinogenesis of human colorectal cancer via an autocrine manner through Smad2/3 pathway. This study provides a novel insight into molecular mechanisms controlling fate of human CCSCs and offers new targets for gene therapy of human colorectal cancer.

## 1. Introduction

Colorectal cancer, also known as colon cancer, results from uncontrolled cell growth in the colon or appendix. Colorectal cancer is the 3rd most common cancer worldwide. There are several treatment options for colorectal cancer, including surgery, chemotherapy, and radiotherapy [1, 2]. In 2008, it has been estimated that 1.23 million new cases of colorectal cancer were diagnosed across the world and, notably, colorectal cancer killed 608,000 patients. Recently, colon cancer stem cells (CCSCs) have attracted considerable attention since they might be potential targets for colon cancer treatment [3].

In 1994, Lapidot et al. first proposed the existence of a cancer stem cell fraction in the context of human leukemia [4]. It has been shown that there is a cancer stem cell subset in a wide variety of solid tumors. Cancer stem cells (CSCs) are relatively resistant to therapy, and they are suggested to be responsible for cancer recurrence and probably metastasis in many tumor systems, including brain [5], prostate [5], pancreatic [6], and melanoma tumors [7]. CSCs represent a novel target for drug discovery for cancer; however, the mechanisms that regulate the self-renewal and multipotency of CSCs remain unclear. It has been suggested that CD44 [8], CD24 [7], and CD133 [9] are hallmarks for colon cancer stem cells (CCSCs). In this study, cell surface markers CD44 and CD24 were thus used to select CCSCs from human colorectal cancer cell lines.

Nodal belongs to the transforming growth factor beta (TGF-*β*) superfamily, and it plays essential roles in regulating embryonic development [10] and cell fate determinations [11, 12]. Nodal has been implicated in promoting proliferation of visceral endodermal cells [13], and it is required for the maintenance of the pluripotency of human embryonic stem (ES) cells and expansion of mouse ES cells [14, 15]. Nodal acts on the Activin receptor type IIB (Actr-IIb) and Activin-like kinase (ALK) 4 or ALK7 [11, 14]. We have recently demonstrated that Nodal signals via the autocrine pathway to promote the proliferation and survival of mouse spermatogonial stem cells [16]. It has been shown that Nodal is involved in breast cancer vascularization and it is related to tumorigenesis and metastasis in numerous cancers, for example, breast cancer, melanoma, ovarian cancer, prostate cancer, endometrial cancer, and pancreatic cancer [9, 17, 18]. Nodal has been suggested to promote development and progression of cancer stem cells and tumors [5], and inhibitors for Nodal receptors ALK4/7 pathway might be used for therapeutic strategy via targeting cancer stem cell. Recently, the effects of Wnt signaling pathway [19, 20] and Notch signaling pathway [21] in colon cancer stem cells have been identified. Notably, it has recently been demonstrated that Nodal is required for self-renewal and tumorigenicity of pancreatic cancer stem cells [6]. However, the expression and potential function of Nodal in colon cancer stem cell remain unknown. In this study, we have for the first time probed the expression of Nodal and its receptors ALK-4, ALK-7, and Actr-IIB in various types of human colorectal cancer cell lines, CD44-negative or CD24-positive and -negative human colorectal cancer cell lines, human colon cancer tissues, and adjacent noncancerous colon tissue. Overall, our results indicate that Nodal facilitates the self-renewal of human colon cancer stem cell via an autocrine Smad2/3 pathway.

## 2. Materials and Methods

### 2.1. Human Colorectal Cancer Cell Lines and Culture

Human colorectal cancer cell lines, including SW480 cells and LOVO cells, were purchased from ATCC (Manassas, VA, USA). HCT116 cell line was a kind gift from Dr. Qinghua Shi, Professor of School of Life Sciences, University of Science and Technology of China. All three cell lines were cultured in RPMI 1640 supplemented with 10% fetal bovine serum (FBS, Gibico), 2 mM L-glutamine (Invitrogen Life Technologies, Carlsbad, CA), and 0.5% penicillin/streptomycin (Invitrogen) at 37°C in a humidified 5% CO_2_ incubator. The cells were passaged when they were grown to 50 to 80% confluence.

### 2.2. RNA Extraction and Reverse Transcription-Polymerase Chain Reaction (RT-PCR)

Total RNA was extracted from SW480 cells, LOVO cells, HCT116 cells, CD44-negative SW480 cells, CD44-negative LOVO cells, and CD24-positive and -negative HCT116 cells in triplicate using Trizol reagent (Invitrogen). DNase I was used to remove any potential genomic DNA contamination. Reverse transcription (RT) was performed in triplicate using oligo (dT) and Superscript II reverse transcriptase (Thermo), and PCR was performed according to the protocol as described previously [22]. The forward and reverse primers of the chosen genes, including Nodal, ALK-4, ALK-7, Actr-IIb, and Gapdh, were designed and listed in Table 1. The PCR reaction started at 94°C for 2 min and was carried out as follows: denaturation at 94°C for 30 sec, annealing at a temperature (Tm) as indicated in Table 1 for 45 sec, and elongation at 72°C for 45 sec. After 35 cycles, the samples were incubated for an additional 5 min at 72°C. PCR products were separated by electrophoresis on 1.2% agarose gels, and the gels were exposed to UV light by chemiluminescence (Chemi-Doc XRS, Bio-Rad, Hercules, CA).

### 2.3. Immunocytochemistry

For immunocytochemistry, SW480 cells, LOVO cells, HCT116 cells, CD24-positive and -negative HCT116 cells, and human colon cancer stem cells cultured without or with Nodal were fixed with 4% paraformaldehyde (PFA) and permeabilized in 0.4% triton-X 100 (Sigma-Aldrich) for 45 min. After washing twice with phosphate-buffered saline (PBS), cells were blocked in 1% BSA for 30 min and followed by incubation with primary antibodies at a dilution with 1 : 100 overnight at 4°C. Primary antibodies included anti-NODAL (Abcam), anti-ALK-4 (Santa Cruz Biotechnology), anti-ALK-7 (Santa Cruz), anti-ACTR-IIB (Santa Cruz), anti-CD24 (Abcam), anti-CD44 (Abcam), anti-phos-Smad2, and anti-phos-Smad3 (Cell Signaling Inc). After three washes with PBS, the cells were incubated with the secondary antibody, including FITC-conjugated or rhodamine-conjugated IgG (Jackson ImmunoResearch Laboratories) at a 1 : 200 dilution for 1 hour at room temperature. DAPI (4′-6-diamidino-2-phenylindole) was used to stain cellular nuclei, and the cells were observed for epifluorescence using a confocal fluorescence microscope (Leica). Immunocytochemistry was performed in triplicate and representative images were presented.

### 2.4. Immunohistochemistry

Colon cancer tissues and reciprocally adjacent noncancerous colon tissues were obtained from Renji Hospital, Shanghai Jiao Tong University School of Medicine. To prepare sections, colon cancer tissues and the matched noncancerous colon tissues were fixed in 4% PFA for 3 hours, dehydrated through a series of graded alcohols, embedded in paraffin at 60°C overnight, and sectioned at 5 *μ*m thickness.

Immunohistochemistry was performed in triplicate in human colon cancer tissues and noncancerous colon tissues using antibodies against NODAL (Abcam), ALK-4 (Santa Cruz), CD24 (Abcam), and CD44 (Abcam) at a dilution of 1 : 200 in these tissues according to the procedure as previously described [23].

### 2.5. Western Blots

HCT116 cells, CD24-negative HCT116 cells, and CD24-positive HCT116 cells cultured without Nodal or with Nodal were lysed with RIPA lysis buffer (Beyotime Institute of Biotechnology). After 30 min lysis on ice, cell lysates were cleared by centrifugation at 12,000 g, and the concentration of protein was measured by BCA kit (Dingguo Company, China). Fifty micrograms of cell lysate from each sample was used for SDS-PAGE (Bio-Rad Laboratories, Richmond, CA), and Western blots were performed in triplicate according to the protocol as described previously [23]. The primary antibody included NODAL (Abcam), phos-Smad2 and phos-Smad3 (Cell Signaling Inc.), and ACTB (Abcam). After extensive washes in PBS, the blots were detected by chemiluminescence (Chemi-Doc XRS, Bio-Rad, Hercules, CA).

### 2.6. Isolation of Colon Cancer Stem Cells

Isolation of CD24- and CD44-positive cells from SW480 cells, LOVO cells, and HCT116 cells was carried out by magnetic activated cell sorting (MACS) in triplicate by MACS microbeads (Miltenyi) using antibodies against CD24 (Abcam) or CD44 (Abcam) according to the procedure described previously [24].

### 2.7. Culture of Colon Cancer Stem Cells and Sphere Formation Assay

CD24- and CD44-positive cells from SW480 cells, LOVO cells, and HCT116 cells were cultured with the conditioned-medium containing serum-free RPMI 1640 supplemented without or with 20 ng/mL Nodal (Peprotech) or with 20 *μ*M SB431542 (Sigma-Aldrich). The cells were subsequently cultured in ultralow attachment 6-well plates (Corning Inc.) at a density of less than 5,000 cells/well. Spheres were collected by gentle centrifugation and dissociated with trypsin-EDTA. The resulting single cells were centrifuged and resuspended in the conditioned medium mentioned above to allow forming spheres. The experiments were performed in triplicate and representative data were presented.

## 3. Results

### 3.1. Expression of Nodal Ligand and Its Receptors in Human Colorectal Cancer Cell Lines

**Figure 1:**
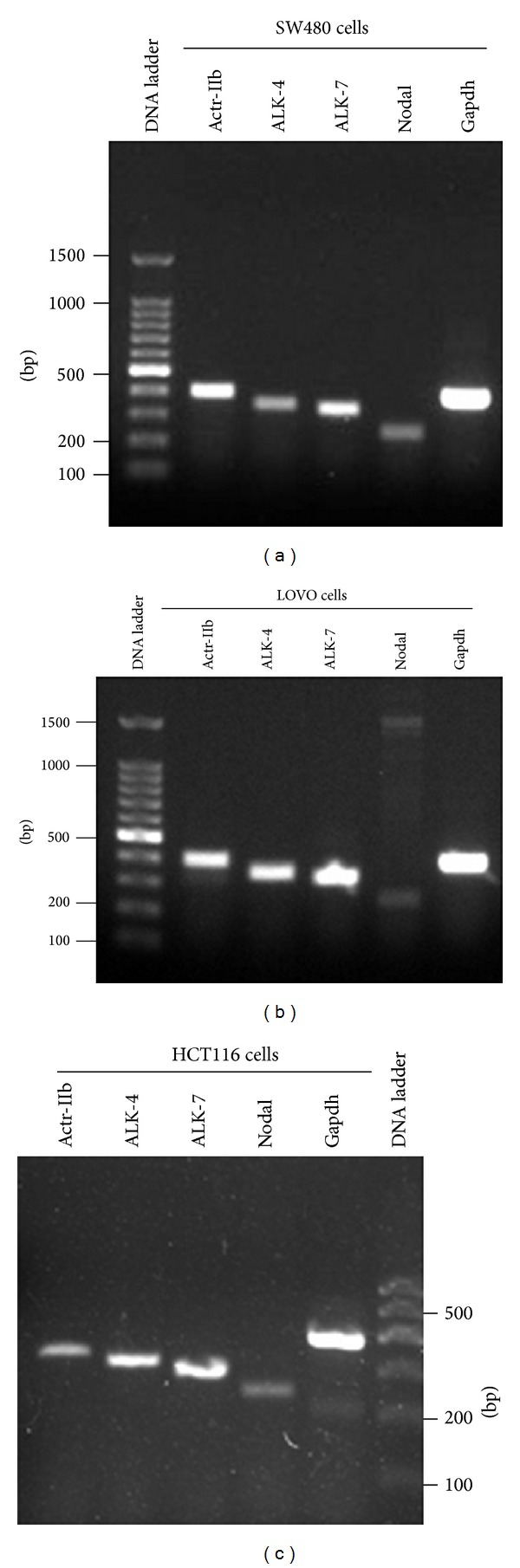
mRNA expression of Nodal ligand and its three receptors in various human colorectal cancer cell lines. (a–c) RT-PCR revealed the transcripts of Nodal, ALK-4, ALK-7, and Actr-IIb in human colon cancer cell lines, including SW480 cells (a), LOVO cells (b), and HCT116 cells (c). Gapdh served as a loading control of total RNA.

**Figure 2:**
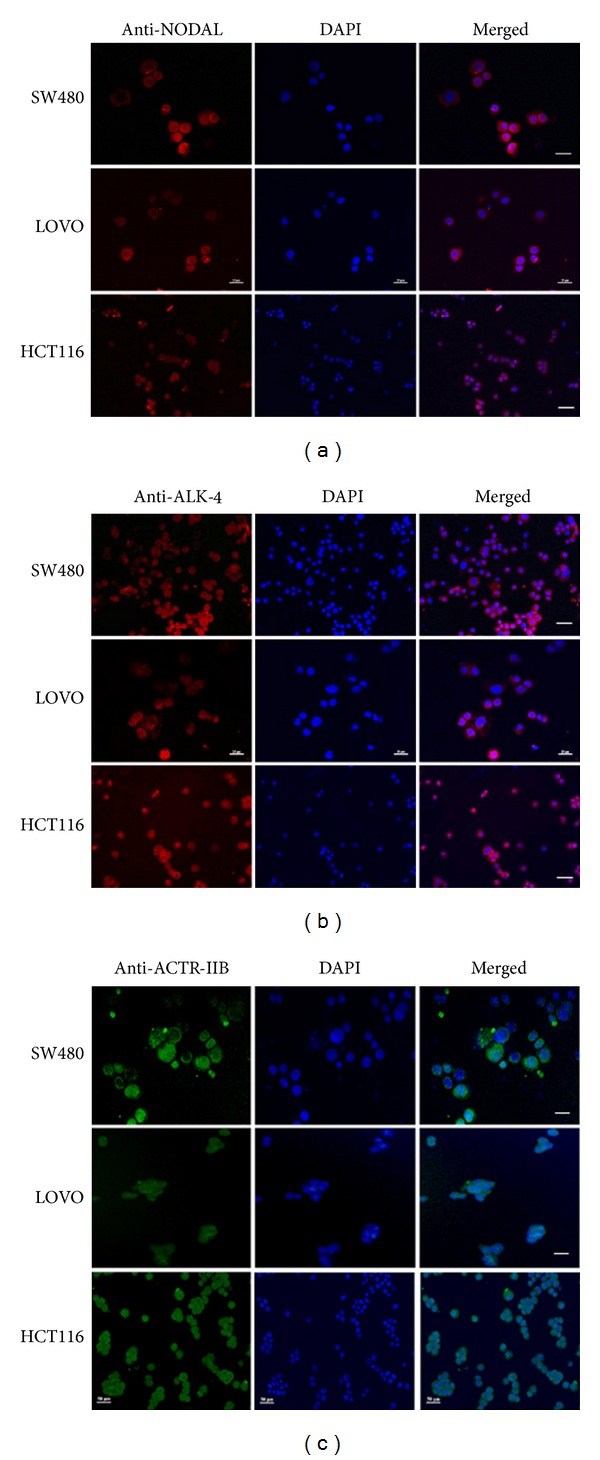
Protein expression of Nodal ligand and its receptors in various human colorectal cancer cell lines. (a–c) Immunocytochemistry showed translation of NODAL (a), ALK-4 (b), and ACTR-IIB (c) in human colon cancer cell lines, including SW480 cells, LOVO cells, and HCT116 cells. Scale bars in (a)–(c) = 10 *μ*m.

We first explored gene expression of Nodal ligand and its three receptors in human colorectal cancer cell lines. RT-PCR showed that the transcripts of Nodal, ALK-4, ALK-7, and Actr-IIb were expressed in SW480 cells (Figure 1(a)), LOVO cells (Figure 1(b)), and HCT116 cells (Figure 1(c)). Immunocytochemistry revealed that the proteins of NODAL (Figure 2(a)), ALK-4 (Figure 2(b)), and ACTR-IIB (Figure 2(c)) were expressed in SW480 cells, LOVO cells, and HCT116 cells. Considered together, these results suggest that both Nodal ligand and its receptors are present in human colorectal cancer cell lines.

### 3.2. Expression of NODAL Ligand and Its Receptor in Human Colon Cancer Tissues and Adjacent Noncancerous Colon Tissues

**Figure 3:**
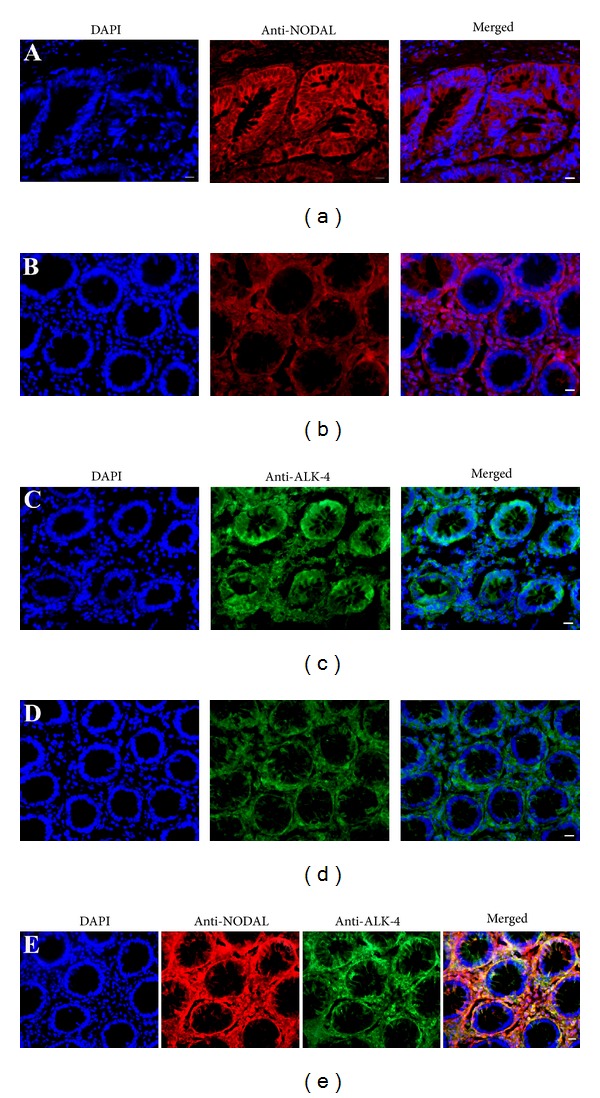
Expression of NODAL ligand and its receptor ALK-4 in human colon cancer tissues and noncancerous colon tissues. (a–d) Immunohistochemistry revealed the expression of NODAL in human colon cancer tissues (a) and adjacent noncancerous colon tissues (b) and ALK-4 in human colon cancer tissues (c) and adjacent noncancerous colon tissues (d), as well as Nodal and its receptor ALK-4 in human colon cancer tissues (e). Scale bars in (a)–(e) = 10 *μ*m.

We next examined the cellular expression pattern of NODAL ligand and its receptor in human colon cancer tissues and adjacent noncancerous colon tissues. Immunohistochemistry displayed that NODAL was expressed at a higher level in human colon cancer tissues than in adjacent noncancerous colon tissues (Figure 3(b)), as evidenced by our observations that 90% of cells in colon cancerous glandular tissues were positive for NODAL (Figure 3(a)), whereas only 30% of cells in colon noncancerous glandular tissues were stained positively for NODAL (Figure 3(b)). Nodal receptor ALK-4 was detected in human colon cancer tissues (Figure 3(c)) and adjacent noncancerous colon tissues (Figure 3(d)). Notably, double staining showed that NODAL and its receptor ALK-4 were coexpressed in human colon cancer tissues (Figure 3(e)), suggesting that Nodal acts as an autocrine pathway in human colon cancer.

### 3.3. Expression of CD44 and CD24 in Human Colon Cancer Tissues and Adjacent Noncancerous Colon Tissues

**Figure 4:**
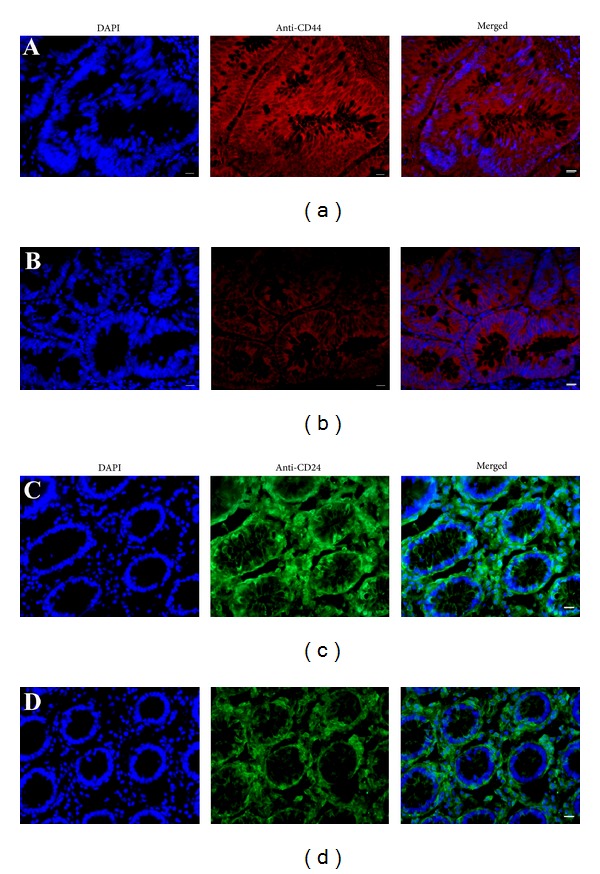
Expression of CD44 and CD24 in human colon cancer tissues and noncancerous colon tissues. (a–d) Immunohistochemistry showed the expression of CD44 in human colon cancer tissues (a) and adjacent noncancerous colon tissues (b) as well as CD24 in human colon cancer tissues (c) and adjacent noncancerous colon tissues (d). Scale bars in (a)–(d) = 10 *μ*m.

CD44 and CD24 have been suggested to be markers for CCSCs [7, 8, 25]. Using immunohistochemistry, we found that CD44 (95%, Figure 4(a)) and CD24 (91%, Figure 4(c)) were expressed abundantly in human colon cancer glandular tissues, whereas CD44 (35%, Figure 4(b)) and CD24 (20%, Figure 4(d)) were undetected or weakly detectable in human adjacent noncancerous colon glandular tissues.

### 3.4. Expression of Nodal Ligand and Its Receptors in CD44- as well as CD24-Negative and -Positive Colon Cancer Cells

**Figure 5:**
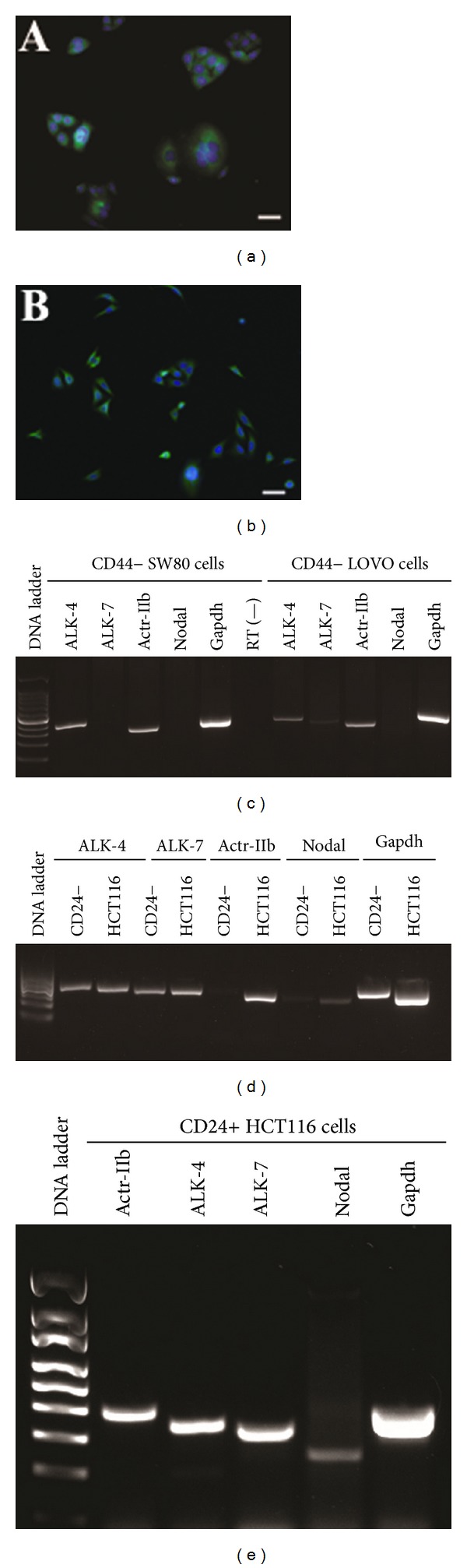
Expression of CD24 and CD44, as well as Nodal and its receptors in CD44- and CD24-positive and -negative colon cancer cells. (a-b) Immunofluorescence showed the expression of CD24 (a) and CD44 (b) in the freshly isolated CD24- and CD44-positive HCT116 cells. (c-d) RT-PCR revealed transcripts of Nodal, ALK-4, ALK-7, and Actr-IIb in CD44-negative SW480 cells and CD44-negative LOVO cells (c) as well as in CD24-negative HCT116 cells (d). Gapdh was used as a loading control of total RNA. (e) RT-PCR displayed transcripts of Nodal, ALK-4, ALK-7, and Actr-IIb in CD24-positive HCT116 cells. Gapdh was used as a loading control of total RNA.

We further probed the expression of Nodal ligand and its receptors in CD44-negative as well as CD24-positive and -negative colon cancer cells. Using MACS, we selected CD44- and CD24-positive and -negative colon cancer cells from SW480 cells, LOVO cells, and HCT116 cells. The purity of the isolated CCSCs by MACS was more than 95%, as evidenced by the immunofluorescent staining showing the high expression of CD24 (Figure 5(a)) and CD44 (Figure 5(b)) in CD24- and CD44-positive HCT116 cells, respectively. Significantly, we found that Nodal transcript is undetected in CD44-negative SW480 cells or in CD44-negative LOVO cells, and it is very weakly expressed in CD24-negative HCT116 cells (Figures 5(c) and 5(d)). Interestingly, Nodal ligand and its receptors including ALK-4, ALK-7, and Actr-IIb were present in CD24-positive HCT116 cells (Figure 5(e)), further implicating that Nodal signals via an autocrine pathway.

**Figure 6:**
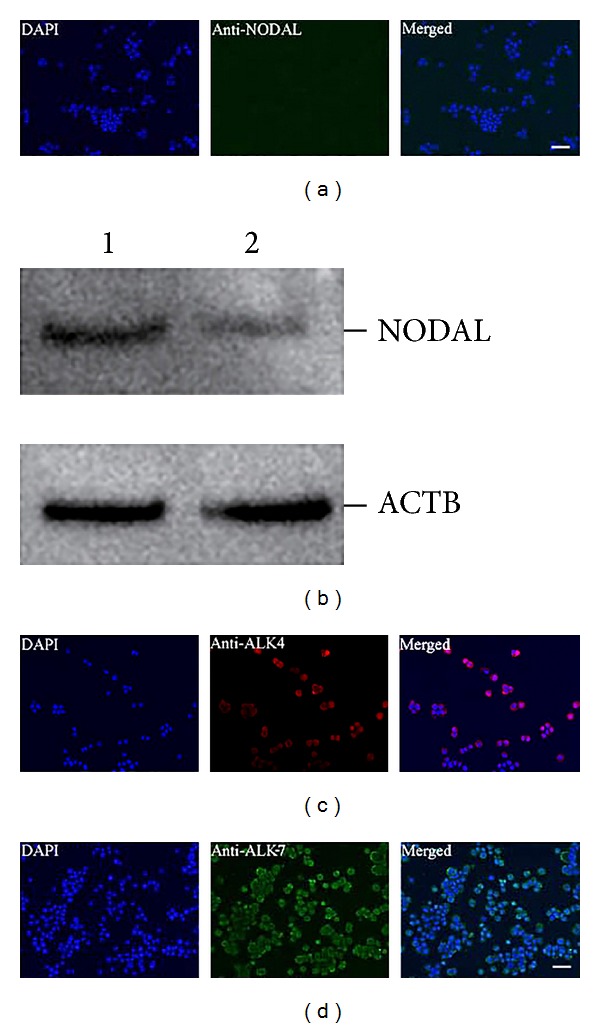
Protein expression of NODAL and its receptors in CD44- and CD24-negative colon cancer cells. (a) Immunocytochemistry showed NODAL translation in CD24-negative HCT116 cells. Scale bars = 20 *μ*m. (b) Western blots displayed NODAL expression in HCT116 cells (lane 1) and CD24-negative HCT116 cells (lane 2). ACTB was used as a loading control of total proteins. (c-d) Immunocytochemistry showed the expression of ALK-4 (c) and ALK-7 (d) in CD24-negative HCT116 cells. Scale bars in (c)-(d) = 20 *μ*m.

Immunocytochemistry further revealed that NODAL protein was undetected in CD24-negative HCT116 cells (Figure 6(a)). Western blots showed that NODAL protein was expressed at a lower level in CD24-negative HCT116 cells than in HCT116 cells (Figure 6(b)). RT-PCR showed that ALK-4 and Actr-IIb transcripts were present in CD44-negative SW480 cells and CD44-negative LOVO cells (Figures 5(c) and 5(d)), while ALK-7 gene transcript was hardly detectable in CD44-negative SW480 cells and CD44-negative LOVO cells (Figure 5(c)). Immunocytochemistry displayed that ALK-4 and ALK-7 proteins were present in CD24-negative HCT116 cells (Figures 6(c) and 6(d)). Taken together, these results implicate that neither Nodal transcript nor protein is present in colon cancer nonstem cells while Nodal receptors are present in these cells.

### 3.5. Nodal Promotes Spheroid Formation of Colon Cancer Stem Cell

**Figure 7:**
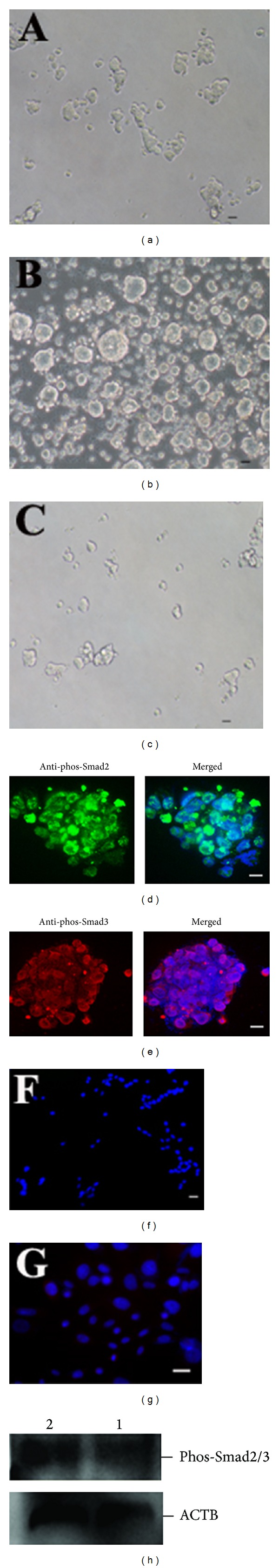
Culture and spheroid formation of CCSCs by Nodal. (a–c) Phase-contrast microscope showed the sphere formation of CCSCs cultured without Nodal (a) or with Nodal (b) or with inhibitor SB431542 (c) for 72 hours. Scale bars in (a)–(c) = 10 *μ*m. (d–g) Immunofluorescence showed the phosphorylation of Smad2 (d) and Smad3 (e) in cells of spheres derived from human CCSCs when cultured with Nodal as well as the phosphorylation of Smad2 (f) and Smad3 (g) in cells of spheres derived from human CCSCs cultured without Nodal. Scale bars in (d), (e), and (g) = 10 *μ*m; bar in *f* = 20 *μ*m. (h) Western blots revealed phos-Smad2/3 expression of in human CCSCs cultured without Nodal (lane 1) and with Nodal (lane 2). ACTB served as a loading control of total proteins.

Cancer stem cells are characterized by their self-renewal as evidenced by the formation of spheres. We next isolated CCSCs from human colorectal cancer cell lines, including SW480 cells, LOVO cells, and HCT116 cells by MACS using antibodies against CD44 and CD24. CCSCs were cultured in serum-free RPMI 1640 supplemented without or with Nodal or with inhibitor SB431542 in 6-well plates with ultralow attachment. Few small cell colonies were observed after culture without Nodal for 72 hours (Figure 7(a)). Notably, more spheres were seen from a single cell colonies after cultures for 72 hours (Figure 7(b)), whereas the size and number of spheres were significantly reduced in CCSCs cultured with Nodal receptor inhibitor SB431542 for 72 hours (Figure 7(c)). Interestingly, immunofluorescence revealed that phosphorylation (phos-) of Smad2 and Smad3 was activated by Nodal in cells of spheres derived from human CCSCs (Figures 7(d) and 7(e)), whereas no signaling was observed in CCSCs in the absence of Nodal (Figures 7(f) and 7(g)). Western blots further verified that phos-Smad2/3 was upregulated by Nodal in human CCSCs (Figure 7(h)), suggesting that Nodal signals via the Smad2/3 pathway.

## 4. Discussion

CSCs are relatively resistant to chemotherapy or radiotherapy and they may be involved in cancer recurrence and metastasis. It has been demonstrated that CSCs play important roles in both tumor initiation and cancer metastasis in many tumor systems, including brain, prostate, pancreatic, and skin tumors [26]. However, in most cases, the underlying mechanisms of CSCs remain largely unclear. Nodal is a member of TGF-*β* superfamily and it is an important regulator for stem cell maintenance, cell proliferation, and differentiation. Recent study has shown that Nodal promotes the self-renewal and tumorigenicity of pancreatic cancer stem cells [6]. Colorectal cancer is one of the most common cancers in the world. Nevertheless, the expression and potential roles of Nodal in colon cancer stem cells need to be clarified.

Nodal signals through activation of a receptor complex, including ALK4, ALK7 and Actr-IIb. Notably, we found, using RT-PCR, immunocytochemistry, and immunohistochemistry, that Nodal ligand and its receptors were present in human colon cancer cell lines (e.g., SW480 cells, LOVO cells, and HCT116 cells) and human colon cancer tissues. Moreover, the expression of Nodal was higher in human colon cancer tissues than that in adjacent noncancerous colon tissues, reflecting that Nodal expression is related to carcinogenesis of human colon cancer. This unique expression pattern of Nodal and its receptors definitively suggests that Nodal signaling is involved in carcinogenesis of human colon cancer via an autocrine manner.

CD24 consists of a small protein core comprising 27 amino acids. It has been reported that cytoplasmic CD24 expression in colorectal cancer is independently correlated with the shortened patient survival [25]. Both CD24 and CD44 have been reported as putative markers for isolating colorectal cancer—initiating cells or CCSCs [7, 8, 25]. We found that CD44 and CD24 were present abundantly in human colon cancer tissues, and conversely, both of them were weakly detectable in human adjacent noncancerous colon tissues. Therefore, CD44 and CD24 could be used as markers for selecting colon cancer stem cells from SW480 cells, LOVO cells, and HCT116 cells by MACS. Significantly, we revealed that Nodal transcript and protein were absent in CD44- and CD24-negative colon cancer cells, whereas it is present in CCSCs, implicating that Nodal is required for maintaining stemness of colon cancer stem cell. Cancer stem cells are capable of self-renewing in that they can form spheres [27]. Interestingly, we found that Nodal promotes spheroid formation of CCSCs via the activation of Smad2 and Smad3 pathways, suggesting that Nodal is involved in self-renewal of colon cancer stem cells. We also observed that NODAL and its receptor ALK-4 were coexpressed in human colon cancer tissues, suggesting that ALK-4 might be involved in the activation of Smad2/3 by Nodal.

In summary, we have for the first time shown the expression of Nodal ligand and its receptors in human colon cancer cell lines as well as in human colon cancer tissues and adjacent noncancerous colon tissues. We have also isolated human CCSCs from human colon cancer cell lines. We highlight that Nodal is involved in self-renewal of colon cancer stem cell via an autocrine manner through the activation of Smad2/3 pathway. This study thus offers a novel insight into carcinogenesis of human colon cancer and molecular mechanisms regulating fate determinations of human colon cancer stem cells. Nodal and its receptors may be used as new targets for treating human colon cancer.

## Figures and Tables

**Table 1:** Primer sequences of genes for RT-PCR.

Genes	Primer sequences	Product size (bp)	Tm (°C)
Nodal	Forward 5′-CCAAGCAGTACAACGCCTAT-3′ Reverse 5′-ACCCACATTCTTCCACGA-3′	234	51

ALK-4	Forward 5′-GCGTGTCTATCACAACCG-3′ Reverse 5′-TCAGCAGCAATAAATCCAA-3′	345	51

ALK-7	Forward 5′-ATAGCGGACTTAGGGTTG-3′ Reverse 5′-CTGGTTTGGGATACTTGG-3′	327	51

Actr-IIb	Forward 5′-AGGCTGAGACACGGGAGT-3′ Reverse 5′-AGGCTGAGACACGGGAGT-3′	394	51

Gapdh	Forward 5′-AATCCCATCACATCTTCC-3′ Reverse 5′-CATCACGCCACAGTTTCC-3′	382	55

## References

[1] Barrier A, Boelle PY, Roser F (2006). Stage II colon cancer prognosis prediction by tumor gene expression profiling. *Journal of Clinical Oncology*.

[2] Merlos-Suárez A, Barriga FM, Jung P (2011). The intestinal stem cell signature identifies colorectal cancer stem cells and predicts disease relapse. *Cell Stem Cell*.

[3] Todaro M, Francipane MG, Medema JP, Stassi G (2010). Colon cancer stem cells: promise of targeted therapy. *Gastroenterology*.

[4] Lapidot T, Sirard C, Vormoor J (1994). A cell initiating human acute myeloid leukaemia after transplantation into SCID mice. *Nature*.

[5] Postovit L, Margaryan NV, Seftor EA (2008). Human embryonic stem cell microenvironment suppresses the tumorigenic phenotype of aggressive cancer cells. *Proceedings of the National Academy of Sciences of the United States of America*.

[6] Lonardo E, Hermann PC, Mueller M (2011). Nodal/activin signaling drives self-renewal and tumorigenicity of pancreatic cancer stem cells and provides a target for combined drug therapy. *Cell Stem Cell*.

[7] Choi D, Lee HW, Hur KY (2009). Cancer stem cell markers CD133 and CD24 correlate with invasiveness and differentiation in colorectal adenocarinoma. *World Journal of Gastroenterology*.

[8] Du L, Wang H, He L (2008). CD44 is of functional importance for colorectal cancer stem cells. *Clinical Cancer Research*.

[9] Quail DF, Walsh LA, Zhang G (2012). Embryonic protein nodal promotes breast cancer vascularization. *Cancer Research*.

[10] Jörnvall H, Reissmann E, Andersson O, Mehrkash M, Ibáñez CF (2004). ALK7, a receptor for nodal, is dispensable for embryogenesis and left-right patterning in the mouse. *Molecular and Cellular Biology*.

[11] James D, Levine AJ, Besser D, Hemmati-Brivanlou A (2005). TGF*β*/activin/nodal signaling is necessary for the maintenance of pluripotency in human embryonic stem cells. *Development*.

[12] Pantic I (2011). Cancer stem cell hypotheses: impact on modern molecular physiology and pharmacology research. *Journal of Biosciences*.

[13] Yamamoto M, Saijoh Y, Perea-Gomez A (2004). Nodal antagonists regulate formation of the anteroposterior axis of the mouse embryo. *Nature*.

[14] Besser D (2004). Expression of nodal, lefty-A, and lefty-B in undifferentiated human embryonic stem cells requires activation of Smad2/3. *The Journal of Biological Chemistry*.

[15] Ogawa K, Saito A, Matsui H (2007). Activin-Nodal signaling is involved in propagation of mouse embryonic stem cells. *Journal of Cell Science*.

[16] Zuping H, Jiji J, Kokkinaki M, Dym M (2009). Nodal signaling via an autocrine pathway promotes proliferation of mouse spermatogonial stem/progenitor cells through Smad2/3 and Oct-4 activation. *Stem Cells*.

[17] Hermann PC, Huber SL, Herrler T (2007). Distinct populations of cancer stem cells determine tumor growth and metastatic activity in human pancreatic cancer. *Cell Stem Cell*.

[18] Papageorgiou I, Nicholls PK, Wang F (2009). Expression of nodal signalling components in cycling human endometrium and in endometrial cancer. *Reproductive Biology and Endocrinology*.

[19] Reya T, Clevers H (2005). Wnt signalling in stem cells and cancer. *Nature*.

[20] Roy S, Majumdar AP (2012). Signaling in colon cancer stem cells. *Journal of Molecular Signaling*.

[21] Strizzi L, Hardy KM, Seftor EA (2009). Development and cancer: at the crossroads of Nodal and Notch signaling. *Cancer Research*.

[22] He Z, Jiang J, Hofmann M, Dym M (2007). Gfra1 silencing in mouse spermatogonial stem cells results in their differentiation via the inactivation of RET tyrosine kinase. *Biology of Reproduction*.

[23] He Z, Jiang J, Kokkinaki M, Golestaneh N, Hofmann M, Dym M (2008). Gdnf upregulates c-fos transcription via the Ras/Wrk1/2 pathway to promote mouse spermatogonial stem cell proliferation. *Stem Cells*.

[24] Kreso A, O’Brien CA (2008). Colon cancer stem cells. *Current Protocols in Stem Cell Biology*.

[25] Wielenga VJM, Heider KH, Offerhaus GJA (1993). Expression of CD44 variant proteins in human colorectal cancer is related to tumor progression. *Cancer Research*.

[26] Todaro M, Alea MP, Di Stefano AB (2007). Colon cancer stem cells dictate tumor growth and resist cell death by production of interleukin-4. *Cell Stem Cell*.

[27] Yeung TM, Gandhi SC, Wilding JL, Muschel R, Bodmer WF (2010). Cancer stem cells from colorectal cancer-derived cell lines. *Proceedings of the National Academy of Sciences of the United States of America*.

